# Effect of RvD1/FPR2 on inflammatory response in chorioamnionitis

**DOI:** 10.1111/jcmm.15963

**Published:** 2020-10-06

**Authors:** Anna Li, Lin Zhang, Junxia Li, Zhenya Fang, Shuxian Li, Yanjie Peng, Meihua Zhang, Xietong Wang

**Affiliations:** ^1^ Key Laboratory of Birth Regulation and Control Technology of National Health Commission of China Maternal and Child Health Care Hospital of Shandong Province Jinan China; ^2^ Department of Occupational and Environmental Hygiene School of Public Health Weifang Medical University Weifang China; ^3^ Department of Obstetrics and Gynecology Provincial Hospital Affiliated to Shandong University Jinan China

**Keywords:** chorioamnionitis, formyl peptide receptor 2, NF‐κB, PPARγ, resolvin D1, trophoblast

## Abstract

Chorioamnionitis (CAM), as a common intrauterine infectious disease, is the leading cause of premature birth, stillbirth, neonatal infection and sepsis. The formyl peptide receptor 2 (FPR2) is a member of GPCRs widely distributed in a variety of tissues and is associated with many inflammatory diseases. With the discovery of FPR2 in human placenta, the possibility of exploring the function of FPR2 in obstetrics is evolving. The Resolvin D1 (RvD1) plays an important role in the resolution of inflammation by combining with FPR2. In this study, we evaluated the role of FPR2 and RvD1 in CAM, not only in the human placenta but also in mouse models. The expression of FPR2 increased in the placenta of CAM patients and the downstream PPARγ/NF‐κB signalling changed accordingly. Moreover, Fpr2^−/−^ mice were highly susceptible to LPS, displaying a worse CAM symptom, compared with WT mice. By establishing a model of trophoblast inflammation in vitro, it was confirmed that RvD1 rescued the effect of LPS on inflammation by combining with FPR2 and its downstream PPARγ/NF‐κB pathway. Otherwise, RvD1 improved the preterm labour in a mouse model of CAM induced by LPS. Altogether, these findings show that RvD1 alleviated the inflammation of trophoblast in vivo and in vitro through FPR2/PPARγ/NF‐κB pathway, suggesting RvD1/FPR2 might be a novel therapeutic strategy to alleviate CAM.

## 1 INTRODUCTION

The incidence of preterm birth (PTB) is about 5%‐18%, but it causes 85% of neonatal death.^1^, ^2^ Intrauterine infection is a major cause of preterm delivery, accounting for 40% of all PTB cases worldwide.^3^, ^4^ Chorioamnionitis (CAM) is a common complication of pregnancy, which is the leading cause of premature delivery, stillbirth, neonatal infection and septicaemia worldwide.^5^ As a specific intrauterine infectious disease, CAM is also closely related to foetal growth and development, occurrence and development of neonatal diseases and so on, which has attracted the attention of obstetricians and paediatrist.^6^, ^7^ Retrograde reproductive tract infection plays an important role in CAM, following the induction of inflammatory responses, with consequent production of cytokines, chemokines and neutrophil recruitment. Moreover, excessive inflammation incurred by limiting infection may also result in tissue injury.^8^ Therefore, inflammation should be modulated to balance the resolution of infection and homeostasis.^9^

The formyl peptide receptor 2 (FPR2) is a member of the G protein‐coupled receptor (GPCRs) family, which plays a crucial role in the resolution of inflammation and interact with multiple exogenous inflammatory ligands, including Annexin A1, LXA4 and Resolvin D1.^10^, ^11^

Resolvin is the ligand of FPR2, which shows an effect on resolving inflammation such as stimulating recruitment of monocytes and apoptosis of neutrophils, regulating neutrophil migration and promoting tissue repair.^12^, ^13^, ^14^ RvD1, as a prominent member of the resolvin, has been proven to regulate PPARγ/NF‐κB signalling pathway after binding with FPR2 and plays a crucial part in cell apoptosis and inflammatory response.^15^, ^16^, ^17^ Although the protective effects of RvD1 have been well characterized in different disease models, few studies have focused on the effects of RvD1 on the obstetric disease, with a particular emphasis on the regulation of placenta inflammation. Thus, we expected to further investigate the effects of RvD1 on trophoblast function in the context of the resolution of placenta inflammation.

In this project, the study was carried out from clinical placental tissue samples to clarify the difference of FPR2 expression in placenta tissue and RvD1 concentration in peripheral blood of CAM women and proposed that FPR2 had an effect on CAM, and the relative deficiency of RvD1 may accelerate the development of inflammation. By using neutrophils derived from Fpr2 gene knockout mice, human embryonic kidney 293 cells (HEK 293 cells) transfected with Fpr2 gene and Fpr2 gene knockout infectious pregnant mice model, to clarify the function and mechanism of Fpr2 in neutrophil chemotaxis and to kill *E coli*, and to explore the protective effect of Fpr2 on pregnancy infection. To confirm the anti‐inflammation effect of RvD1 on trophoblast, the inflammation model was induced by LPS through combining with FPR2 in vitro. To explore the therapeutic effect of RvD1/FPR2 on pregnant infectious diseases, we further observed the effect of RvD1 application on infectious pregnant mice model.

## 2 MATERIALS AND METHODS

### 2.1 Patient and sample collection

Clinical samples were obtained from Maternal and Child health care Hospital of Shandong Province, Shandong University in accordance to relevant ethical regulations for clinical specimen research, with the protocols approved by the Ethics Committee of Shandong University. Written informed consent was obtained from all patients.

Peripheral blood and placental tissues were selected from patients with pregnancy ≥37 weeks, premature rupture of membranes, caesarean delivery in our hospital, CAM diagnosed definitely by placental pathology or spontaneous preterm birth (PTB) without histologic chorioamnionitis, as the control (CT). All the participants met the standard without pre‐eclampsia, hypertension, multiple gestation, asthma, cardiovascular diseases, cervical incompetence, diabetes, foetal growth restriction, foetal malformation, sexually transmitted diseases, thyroid disease and uterine malformations.

### 2.2 Mice maintenance and drug administration

Fpr2^−/−^ mice were generated from Jiming Wang's laboratory of the National Institute of Health with C57BL/6 genetic background,^18^ and WT C57BL/6 mice were used as control, which were maintained in SPF conditions with the temperature at 20‐25°C and 12‐hour cycles of light and night. Animal experiments were carried out in accordance with relevant ethical regulations for animal research. The protocol was approved by the Institutional Animal Care and Use Committee of Shandong University.

Pregnancy mice, 6‐8 weeks old, were obtained by mating with males at night, which were determined by the vaginal plug next morning, designated as day 0.5 of pregnancy. The pregnant mice infection model was established by intraperitoneal injection of LPS 0.2 mg/kg at D15 of pregnancy and 0.6 mg/kg after 3 hours.^19^

To investigate the effect of RvD1, WT pregnant mice were divided into four groups (CT, LPS, RvD1, LPS + RvD1). CT group: intraperitoneal injection of PBS; LPS group: injection of LPS at a dose of 0.2 mg/kg, 3 hours later repeat the injection at a dose of 0.6 mg/kg at D15 of pregnancy; RvD1 group: RvD1 was injected intravenously into the tail at a dose of 10 µg/kg at D15 of pregnancy; LPS + RvD1 group: 24 hours and 30 minutes before the first injection of LPS at 15D of pregnancy, injected with RvD1 at a dose of 10 µg/kg through the tail vein. Four hours after the final injection, the mice were killed for further testing.

### 2.3 Cell culture

Human trophoblast cell line HTR8/Svneo were purchased from the ATCC (American). Cells were cultured in DMEM supplemented with 10% foetal bovine serum, 1% penicillin and 1% streptomycin and incubated in an incubator at 37°C with 5% CO_2_. Cells were divided into four groups: CT group: treated with PBS; LPS group: treat with 10 µg/mL LPS; LPS + RvD1 group: treated with 150 nmol/L RvD1 30 minutes before LPS; LPS + RvD1 + WRW4(L + R + W) group: treated with 150 nmol/L RvD1 and 10 µg/mL WRW4 30 minutes before LPS. Eight hours later, cells were collected for the next steps.

### 2.4 Histology and immunochemistry

Placenta of patients was collected immediately after caesarean and washed with PBS, with one part was stored in liquid nitrogen, and other parts were fixed in 4% paraformaldehyde, embedded in paraffin, sectioned and stained with haematoxylin and eosin. For immunostaining, slices were boiled in 0.01 mol/L citrate buffer (PH6.0) to retrieve antigen and then naturally cooled to room temperature. Methanol containing 3% H_2_O_2_ was used to eliminate endogenous peroxidases. Before blocked in 10% goat serum for 30 minutes, the tissue was permeabilized with 0.1% Triton X‐100 for 30 minutes. The sections were incubated with primary antibodies (anti‐FPR2, anti‐5‐LOX, anti‐15‐LOX from Abcam; anti‐PPARγ, anti‐P65 from Santa Cruz) overnight at 4°C, incubated with the secondary antibody at 37°C for 60 minutes and developed with diaminobenzidine tetrahydrochloride. For cell culture slides, secondary antibodies conjugated to fluorescein isothiocyanate (Sigma‐Aldrich) were used.

### 2.5 Western blotting

Placenta tissues were cut into small pieces and homogenized by Potter‐Elvehjem Tissue Grinders on ice, followed by lysed in the RIPA buffer containing 1 mmol/L PMSF, as well as the cells. After quantification by BCA assay, 10 µg protein of each sample was resolved by polyacrylamide gel electrophoresis and transferred to PVDF membranes. Blots were blocked by 5% no‐fat milk for 1 hour and then incubated with specific primary antibodies (anti‐FPR2, anti‐5‐LOX, anti‐15‐LOX from Abcam; anti‐PPARγ, anti‐P65 from Santa Cruz, anti‐IκBα from Servicebio) overnight at 4°C. Blots were incubated with HRP‐conjugated secondary antibody and developed using the ECL system. The protein bands were acquired and quantitated using the Amersham Imager 600(GE) blot documentation instrument provided by the FluoChem M system (Protein Simple).

### 2.6 Quantitative PCR

Total RNA was extracted from placenta tissues and cells with TRIzol reagent according to the manufacturer's protocol and then converted to complementary DNA using First Strand cDNA Synthesis Kit. The target mRNA was normalized to the levels of β‐actin, which was detected and quantified by real‐time PCR using the Applied Biosystems 7500 system. The following primers were used in this study: *FPR2* forward primer: 5′‐ATGTCCATTGTTGCCATCTGC‐3′, reverse primer: 5′‐GACGTAAAGCATGGGGTTGAG‐3′; GAPDH forward primer: 5′‐CTCATACATAAAGTCCTTCCCGC‐3′, reverse primer: 5′‐GGCGGTTGATTTGTCTGTTGT‐3′.

### 2.7 Statistics

All data were analysed and visualized using GraphPad Prism version 8.0. The replicated data are expressed as the mean with the standard error of the mean. The number of samples (n) is indicated in the figure legends. The differences between the two groups were compared using unpaired two‐tailed Student's *t* test. A *P*‐value < .05 was considered as statistically significant unless otherwise indicated. 1

## 3 RESULTS

### 3.1 The expression of FPR2 significantly increased in placental tissue from CAM

**FIGURE 1:**
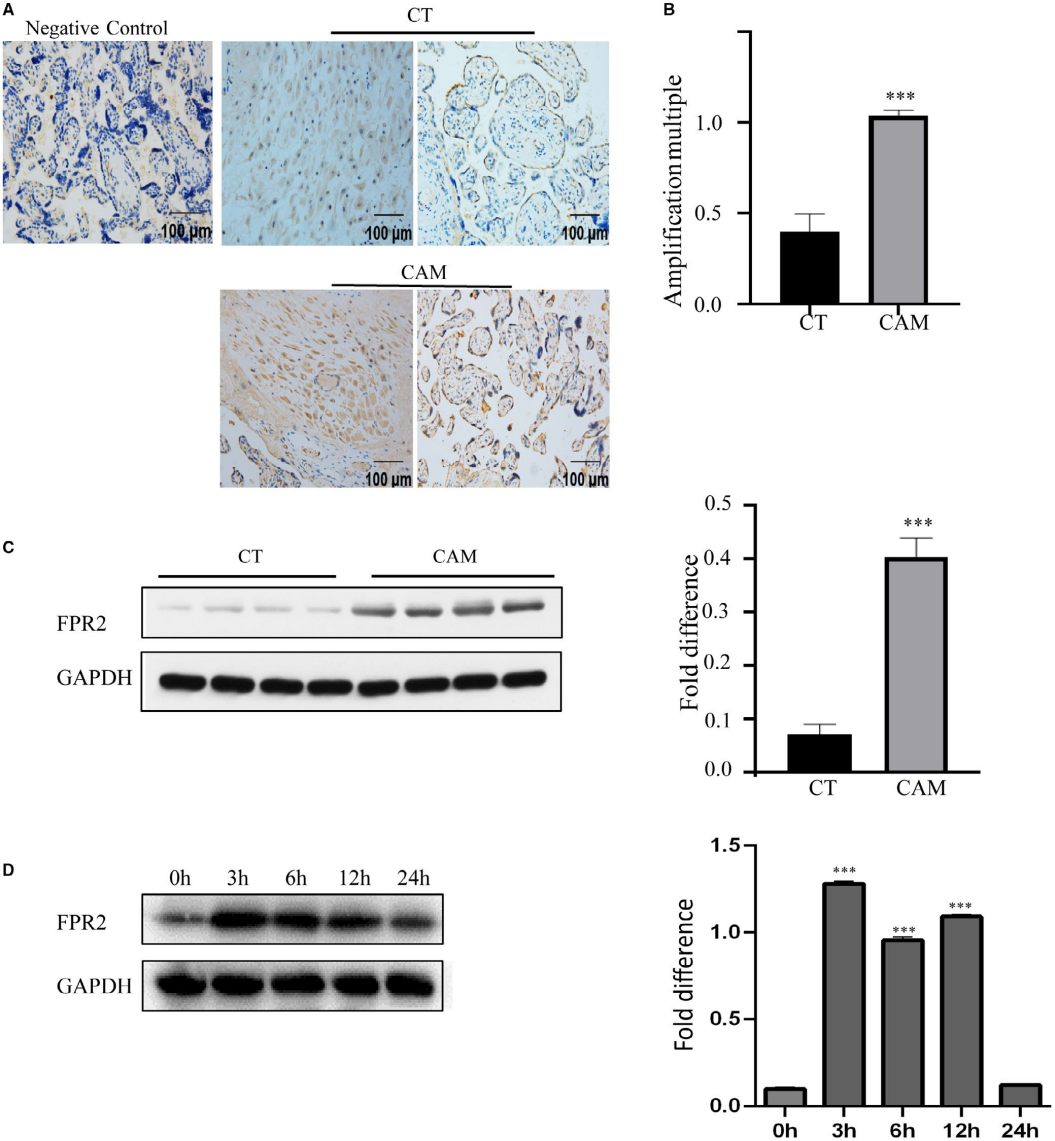
Expression of FPR2 increased in the placenta of CAM patients. A, The expression of FPR2 in the placenta from CT (n = 10) and CAM patient (n = 10) were detected by immunohistochemistry, Scale bar, 100 µm. B, The mRNA expression of FPR2 in the placenta was analysed by qPCR. C, Expression of FPR2 in human placental tissues from CAM patients and in preterm control (CT) was determined by Western blotting analysis. D, HTR8 cell were treated with LPS (100 µg/mL) for different periods of time and then harvested for Western blot analysis of the expression of FPR2. Data are shown as mean ± SEM Statistics: *P < .05, **P < .05, ***P < .001 (n = 6)

To confirm the relationship between FPR2 and CAM, we detected the location and expression of FPR2 in the placenta of women with or without CAM, about 37 weeks of pregnancy. As shown in Figure 1A, immunohistochemical analysis of the paraffin‐embedded placenta was performed to investigate the location and expression of FPR2 in the placenta. Fpr2 mainly located on cytotrophoblast and decidual cells in both groups. Meanwhile, the expression of FPR2 in the placenta with CAM, compared with the control group, increased significantly. In order to further prove the increase of FPR2 in CAM placenta, we performed qPCR and Western blotting using placental tissues from both CT and CAM group. As shown in Figure 1B (right), the mRNA expression of FPR2 was strikingly up‐regulated in women with CAM. Similar changes of placental FPR2 at the protein level were also confirmed by Western blotting (Figure 1C). These quantitative results (Figure 1C right) support the immunohistochemical data presented in Figure 1A. Furthermore, we observed the change of FPR2 in response to LPS in the HTR8 cell line, which is used to provoke inflammation. The result showed that FPR2 increased significantly from 3 to 12 hours after treating with LPS. Therefore, the up‐regulation of FPR2 is correlated with CAM (Figure 1D).

### 3.2 FPR2 absence aggravated the symptoms of CAM

**FIGURE 2:**
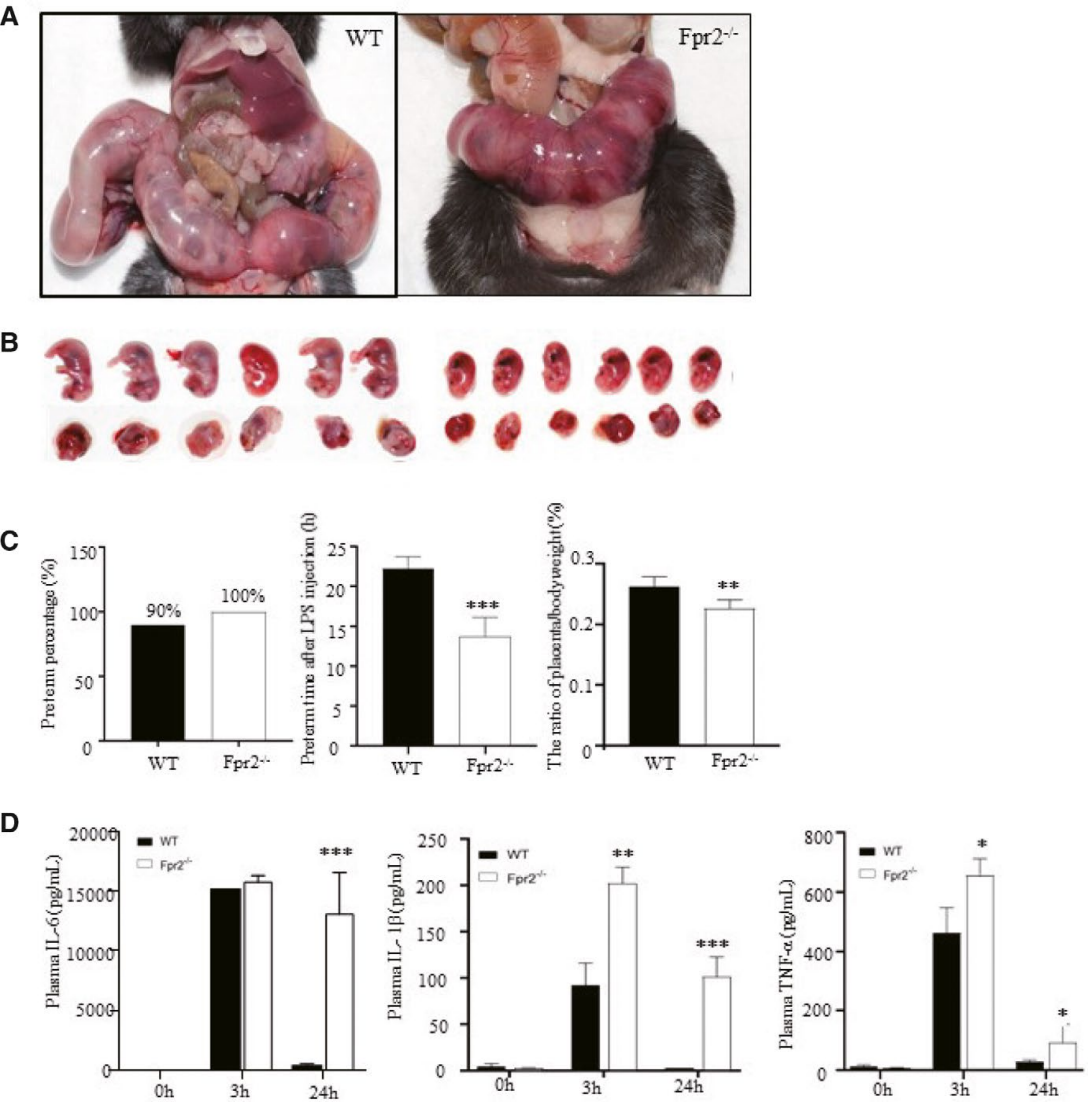
FPR2 absence aggravated the symptoms of mice model of CAM induced by LPS. A, Representative photographs of uterus morphology of WT and Fpr2−/− mouse injected with LPS on the 15th day of pregnancy to induced preterm. FPR2 absence exacerbates the deleterious effects of LPS on uterus morphology. B, Infarction of placenta and foetuses. C, FPR2 absence shortens the preterm time after LPS injection and reduces the weight of the placenta, but the preterm percentage did not change. D, The inflammatory factors in serum were measured by ELISA. N = 5 mice per group. Data are shown as mean ± SEM Statistics:*P < .05, **P < .05, ***P < .001

Next, we evaluated whether the absence of FPR2 would affect the development of CAM. Given this, WT and Fpr2^−/−^ pregnant mice were induced by LPS with two consecutive i.p. injections to induce preterm labour. Preterm delivery time and placenta weight after LPS injection was detected. Also, systemic inflammation and placental inflammation were evaluated. Fpr2^−/−^ mice were significantly more susceptible to LPS with shorter preterm time after LPS injection. Compared with WT mice, the placenta and foetus of Fpr2^−/−^ mice were smaller and less mature (Figure 2B, C). Congestion of uterus and placenta was more apparent in Fpr2^−/−^ pregnant mice with obvious necrotic areas in the placenta (Figure 2A). In addition to the intense local inflammation, inflammatory factors in plasma significantly increased 3 hours after LPS injection (Figure 2D). There were greater amounts of all tested cytokines in the plasma of Fpr2^−/−^ mice. The levels of IL‐6, IL‐1β and TNFɑ were higher in Fpr2^−/−^ mice and similar IL‐10 levels. In general, Fpr2^−/−^ mice exhibited significantly worse CAM symptoms.

### 3.3 FPR2 participated in the process of CAM through PPARγ/NF‐κB pathway

**FIGURE 3:**
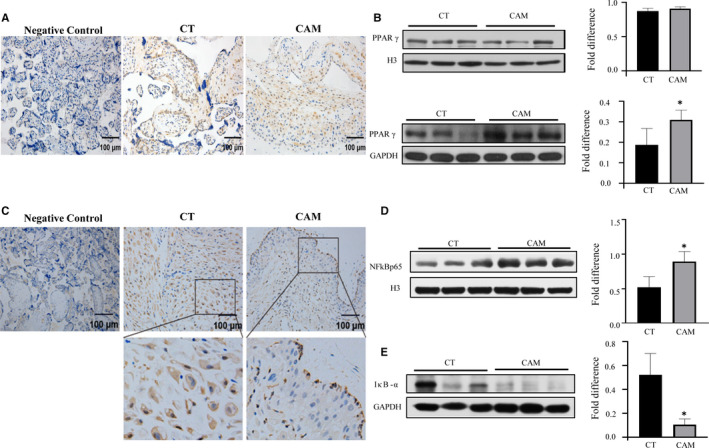
Correlation of FPR2 expression level and downstream member's expression level of PPARγ/NF‑κB signalling in clinical samples. A, The expression of PPARγ in the placenta from CT (n = 10) and CAM patient (n = 10) was detected by immunohistochemistry, Scale bar, 100 µm. B, The expression of PPARγ in nuclear (upper) and cytoplasm (under) was detected by WB. (*P < .05) (C) The expression of P65 in placenta from CT (n = 10) and CAM patient (n = 10) was detected by immunohistochemistry, Scale bar, 100 µm. D, The expression of P65 and IκB‐α was detected by WB. Data are shown as mean ± SEM Statistics: *P < .05, **P < .05, ***P < .001 (n = 6)

To address the question of how FPR2 plays a role in women with CAM, we detected the location and expression of its downstream target proteins. It was reported that FPR2 was closely associated with inflammation and pregnancy immunity adaptation through NF‐κB pathway.^20^, ^21^ Accordingly, we hypothesized that FPR2 participated in the process of CAM through PPARγ/NF‐κB pathway. We firstly examined the location and expression of FPR2 in the placenta from CT and CAM patients. According to immunohistology, PPARγ was mainly distributed in cytotrophoblast and decidual cells, exhibiting cytoplasm and nuclear staining (Figure 3A). As illustrated by Western blotting analysis, the amount of PPARγ in cytoplasm increased in the CAM. However, nuclear transplantation was unchanged (Figure 3B). Moreover, the level of IκB‐α in placenta decreased in CAM patients when compared with the control group (Figure 3E), while the entry of NF‐κB subunit p65 to nuclear increased (Figure 3D), which was consistent with the immunohistochemical analysis. These results further supported the hypothesis that FPR2 could participate in the process of CAM via the PPARγ/NF‐κB pathway. 2

### 3.4 RvD1 diminished LPS‐induced inflammation in trophoblast inflammation model in vitro

**FIGURE 4:**
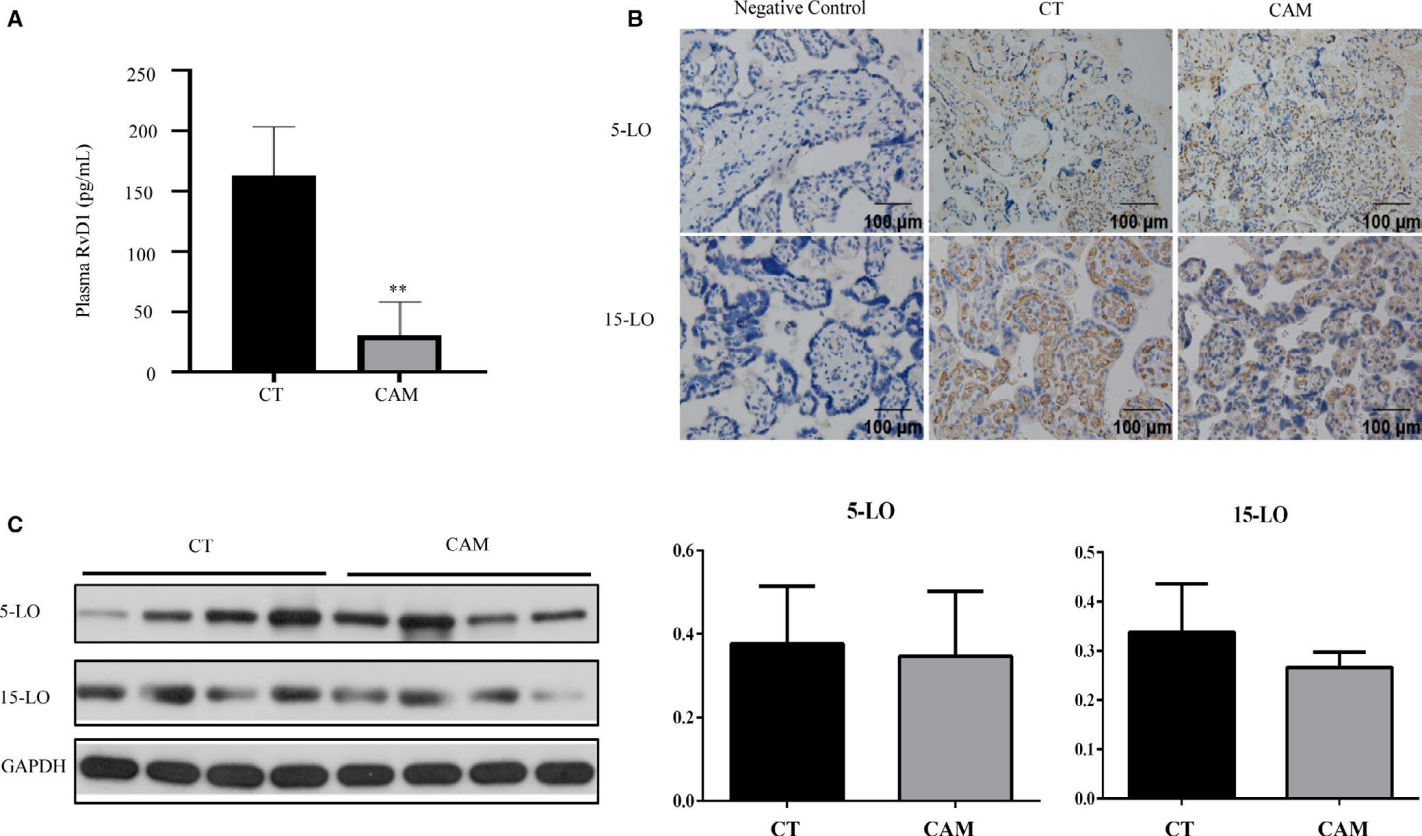
The decrease of RvD1 was not related to the synthesizing. A, The level of RvD1 expression was measured by ELISA. The expression of 5‐LOX and 15‐LOX was assessed by (B) Immunostaining in placenta tissue and (C) WB. Data are shown as mean ± SEM Statistics: *P < .05, **P < .05, ***P < .001 (n = 6)

Now that the effect of FPR2 in the CAM had been illustrated, the focus was turned to RvD1, the ligand of FPR2, which played an important role in many inflammation‐related diseases. Samples derived from CAM patients showed a significant decrease of RvD1 in plasma, compared with the CT group (Figure 4A). Docosahexaenoic acid formed the starting point for RvD1 biosynthesis and then was catalysed by neutrophil‐derived 5‐LO/platelet 5‐LO.^22^, ^23^ In order to explore the cause of RvD1 reduction, we detected the expression of 5‐LO, 12‐LO and 15‐LO in the placenta. As shown in Figure 4B, the 5‐LO and 15‐LO were distributed mainly in syncytiotrophoblast cells. There was no significant change in the expressions of 5‐LO and 15‐LO in the placenta of CAM patients by immunohistochemical staining (Figure 4B). A similar result of placental 5‐LO and 15‐LO at the protein levels were also confirmed by WB (Figure 4C). Therefore, the synthesizing may not be the reason for the down‐regulation of RvD1 in women with CAM.

**FIGURE 5:**
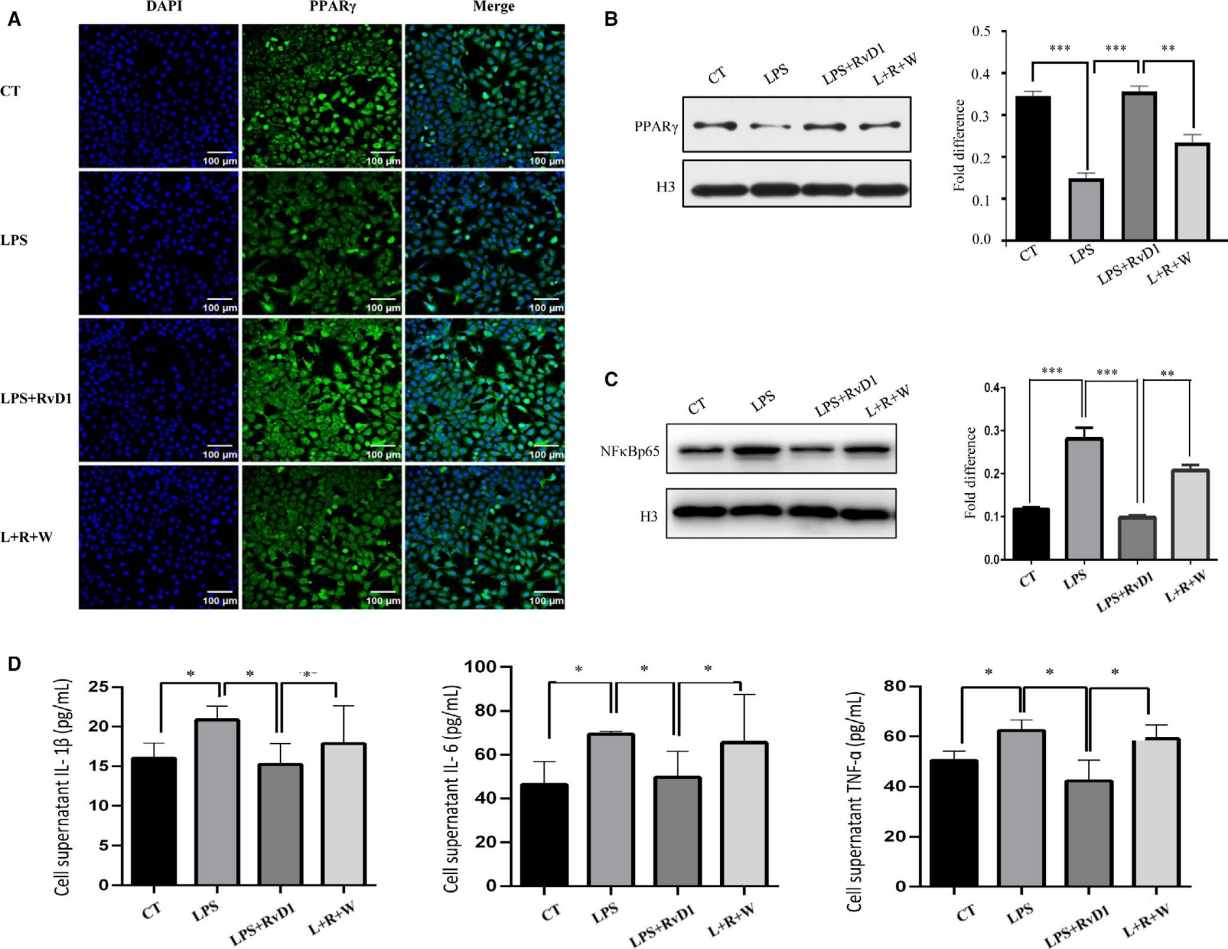
RvD1 resolved inflammation in trophoblast through FPR2/PPARγ/NF‐κB pathway. HTR8 cells were divided into 4 groups, treated with PBS, LPS, LPS + RvD1, LPS + RvD1 + WRW4, respectively. PPAR protein level and nuclear translocation were detected by (A) immunocytochemistry and (B) WB. C, The expression of NF‐κBp65 in nuclear was examined by WB. C, The level of IL‐1β, IL‐6, IL‐10, and TNFα in cell supernatant. Data are shown as mean ± SEM Statistics: *P < .05, **P < .05, ***P < .001 (n = 6)

In vitro study, trophoblast cell line HTR8 cells were cultured, and LPS was used to establish a cell model of inflammation to investigate the protective effect of RvD1. We further detected the expression of PPARγ/NF‐κB signalling in response to RvD1. In order to understand the molecular mechanisms involved in the protective effects of Rvd1 against LPS‐induced inflammation, FPR2 antagonist was used at the same time. According to Immunocytochemistry and WB, the nuclear translocation of PPARγ decreased under LPS stimulation and recovered by RvD1, and then the effect of RvD1 was reversed by WRW4 (Figure 5A,B).^24^ Similarly, activation of P65 marked by LPS‐induced nuclear entry was hindered by RvD1, which was abolished by the addition of WRW4 (Figure 5C). To investigate inflammation changes in HTR8 cells treated with LPS and RvD1, or in addition to WRW4, we performed ELISA to detect the inflammatory factors in the supernatant. The production of IL‐1β, IL‐6, IL‐10 and TNF‐α stimulated by LPS was blocked by RvD1, which was recovered by WRW4 (Figure 5D). In general, RvD1 inhibited proinflammatory cytokine expression dependent on activating FPR2. These results indicated that RvD1 resolving inflammation in trophoblast through FPR2/PPARγ/NF‐κB pathway.

### 3.5 RvD1 improved the preterm delivery in the vitro CAM model

**FIGURE 6:**
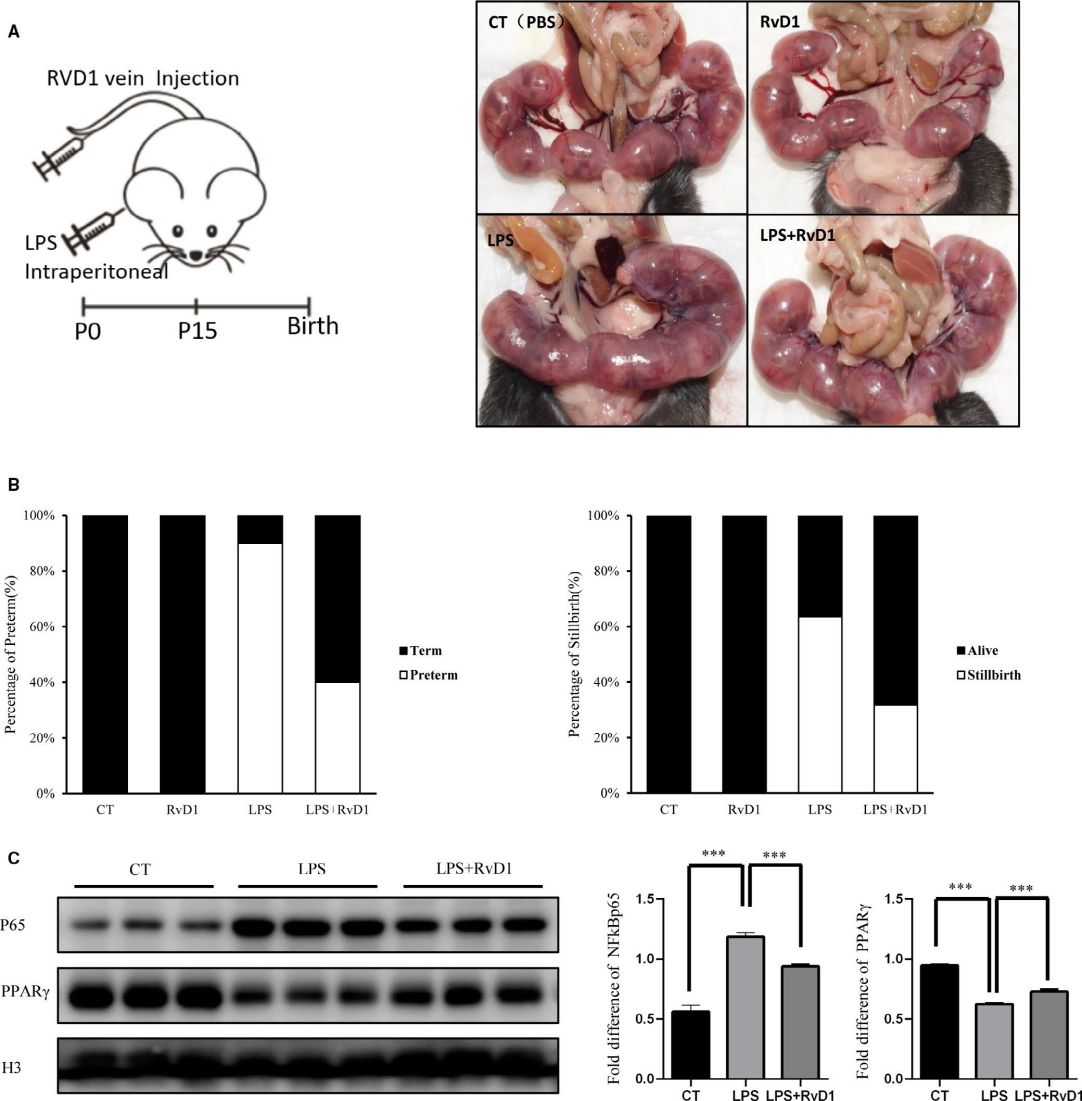
RvD1 improved the preterm labour in an in the Vitro CAM model. The pregnant mice were treated with PBS, LPS, RvD1 and LPS + RvD1, respectively. A, Representative photographs of uterus morphology. B, Percentage of preterm and stillbirth and pup mortality. N = 5 mice per group. C, NF‐κB and PPARγprotein levels in the placenta were detected by WB. N = 3 mice per group. Data are shown as mean ± SEM Statistics: *P < .05, **P < .05, ***P < .001

To study the effects of RvD1 on LPS‐induced CAM, 15‐day pregnant C57BL6 mice were induced preterm delivery, as described previously. Exposure to LPS‐induced preterm delivery in 90% of mice, while cotreated with RvD1, prevented the preterm delivery in 40% of the cases (Figure 6B). Next, the stillbirth rate and pup mortality rate of four groups of mice were assessed. LPS resulted in a stillbirth rate of 64%, while RvD1 reduced pup mortality to 32% (Figure 6B). Moreover, compared with mice treated with PBS, the uterus of the mice treated with LPS showed distinct oedema, loss of inter‐site definition and inflammation, whereas, RvD1 gave relief (Figure 6A). Furthermore, RvD1 reversed the changes of PPARγ and NF‐κB induced by LPS notably in the mice (Figure 6C), which provided corroboration of the relation between FPR2 and PPARγ/NF‐κB.

## 4 DISCUSSION

When the pathogens could not be eliminated timely, Toll‐like receptors (TLRs) on immune cells and trophoblasts were triggered excessively to further activate downstream signalling such as MAPK, NF‐κB and STAT, stimulating the release of inflammatory factors such as IL‐1α, IL‐6, IL‐1β and TNF‐α, promoting the occurrence and expansion of inflammatory reactions.^25^ Once inflammation spread to foetal blood vessels, the foetal infections would bring about premature birth, neonatal infection, even neonatal death. Besides eradicate the pathogen, timely resolution of inflammation is an essential way for avoiding further injury and restoring homeostasis following infection. However, there are no effective measures to determine the pathogen of CAM immediately in the clinic, and it is difficult to predict CAM before clinical symptoms develop.

Several clinical trials confirmed the effect of fish oil in reducing inflammation, which is rich in long‐chain omega‐3 polyunsaturated fatty acids.^26^, ^27^ As a derivative of ω‐3 polyunsaturated fatty acid, RvD1 and its receptor FPR2 had been proved to protect against inflammation in several tissues.^28^, ^29^, ^30^ However, their roles in controlling the inflammatory response in CAM have not yet been explored. In current research, we aimed at elucidating the protective roles of RvD1/FPR2 on trophoblasts, explored its signalling pathway at the same time, in order to provide a new therapeutic method for infectious diseases of pregnancy.

In the present study, we demonstrated that RvD1 decreased in peripheral blood while FPR2 increased in placenta of CAM patients, which were accompanied by inhibition of the nuclear translocation of PPARγ and NF‐κBp65. Moreover, we found that Fpr2 deficiency worsens the inflammatory response of pregnancy mice induced by LPS and exogenous RvD1 administration alleviated damage of placenta and attenuated inflammation in mice model of CAM. In addition, using antagonists of FPR2 and PPARγ could block the rescued effect of RvD1 on trophoblast in vitro, we were able to determine that RvD1, by binding with FPR2, exerted its anti‐inflammatory effects through PPARγ/NF‐κB pathway. These findings indicated that RvD1 administration improved outcomes of CAM at least in part by attenuating inflammation via binding with FPR2 and then interacting with PPARγ/NF‐κB signalling pathway. Our current study showed a decrease of serum RvD1 in patients with CAM, which is consistent with recent studies that RvD1 levels were lowered in patients with systemic lupus erythematosus (SLE) or Rheumatoid arthritis compared with healthy individuals.^31^, ^32^ RvD1 is enzymatically biosynthesized from ω‐3 fatty docosahexaenoic acids (DHA) by different lipoxygenases (LO). We found that no change in 5‐LO and 15‐LO expression in placental tissue, which indicated there was no dysfunction in the synthesization of RvD1. Based on these results, we speculated that the increased consumption of RvD1 in the inflammatory state and the relatively insufficient supply may be one of the development factors of CAM.

We observed the typical characteristics of inflammatory reactions in vivo, such as a large number of neutrophil infiltration in the placental tissue of CAM patients (Figure S1A), an increase of serum inflammatory factors (Figure S1B). By the way, IL‐10 also increased significantly in CAM. The possible reason is that the basic function of IL‐10 is to reduce cytokines produced by TH1 cells, which play a role in immunosuppression with anti‐inflammatory activity, and cannot reflect the degree of infection in the early stages of inflammation.^26^, ^33^ FPR2 regulates inflammatory response in the human decidua and endometrium of early pregnancy, as well as inflammatory events in physiological and pathological delivery.^27^ It was reported that the expression of FPR2 decreased in the peripheral blood and placentae of women and rat model of pre‐eclampsia, accompanied by increased inflammation, indicating that down‐regulation of FPR2/Fpr2 contributed to peripheral and placental inflammation in PE pregnancies.^34^ In FGR, enhanced inflammation is also associated with inadequate placental development and dysfunction.^35^ In addition, the contribution of placental inflammation induced by FPR2 to the pathophysiology of FGR had been investigated. However, the increased expression of FPR2 was found in some diseases, such as hepatic inflammation, autoimmune encephalomyelitis, neonatal hypoxia‐ischaemic injury.^36^, ^37^, ^38^ The current study showed that the expression of FPR2 in placental tissues of CAM patients, who were carefully selected using strict clinical standards and underlying pathology, was significantly higher than PTB women without CAM. The expression of FPR2 was first found in neutrophils,^39^ and the increased expression of placental FPR2 in CAM patients may be related to the increase of the chemotaxis of neutrophils to the placenta. Furthermore, our result showed that FPR2 expressed in all layers of placenta, including syncytiotrophoblast (ST) and decidua cells (DC), agrees with the recent studies which found that FPR2 expressed in human placental trophoblasts.^40^ Therefore, increased expression of placental FPR2 may also be related to the up‐regulation in trophoblast in inflammatory states. In general, the increased FPR2 may be an effort to strengthen its function under CAM. Meanwhile, we found that Fpr2 deficiency worsens the inflammatory response of pregnancy mice induced by LPS, which suggested a beneficial role of FPR2. Based on the results of this part of the research, we proposed the reduction of FPR2 was related to the development of CAM. The rapid recruitment of neutrophils and the expression of FPR2 may play an important role in destroying and limiting pathogens in the early stage of CAM.

PPARγ, a downstream regulator of FPR2, is a member of nuclear receptor transcription factor superfamily and is involved in a variety of physiological and pathological processes such as lipid and glucose metabolism, energy homeostasis, inflammation, cell proliferation and differentiation. The previous study revealed that PPARγ was involved in the invasion and differentiation of trophoblasts^41^ and played an important role in embryo implantation, placenta formation and trophoblast differentiation and maturation. PPARγ can also inhibit inflammation by entering the nucleus, following binding to NF‐κBp65 and inhibiting its transcriptional activity.^42^ We found that the increased expression of PPARγ in placental tissue cells of patients with CAM may be the stress reaction to inflammation stimuli. However, it was interesting that the expression of PPARγ in nuclear did not change significantly, which suggested that the transport of PPARγ from the cytoplasm to the nucleus may be inhibited. The mechanism of PPARγ in the occurrence and development of CAM will be further elucidated in future studies.

Nuclear factor κB (NF‐κB) is an important inflammatory transcription factor. The activation process of the NF‐κB pathway is phosphorylation and degradation of the NF‐κB inhibitor IκB, and NF‐κBp65 is released into the nucleus to activate downstream genes. In this study, we found that the expression of NF‐κBp65 in CAM patients was significantly up‐regulated, and IκB degradation, indicating that NF‐κB signalling was activated in the placenta of CAM patients. Activation of PPARγ and inhibition of the NF‐κB pathway may be the mechanism by which FPR2/RvD1 plays its pivotal roles.

Bacterial lipopolysaccharide (LPS) is mainly a component of the outer wall of Gram‐negative bacteria, which was used to establish pregnancy‐related inflammatory disease models, such as recurrent miscarriage, premature delivery, intrauterine growth restriction and preeclampsia.^43^, ^44^, ^45^ We found the secretion of inflammatory factors in trophoblast supernatant treated with RvD1 decreased. The application of FPR2 blocker WRW4 could block the activity of RvD1 in inhibiting inflammation, suggesting that the inhibitory effect of RvD1 in the trophoblast inflammation model depended on its combination with FPR2. We also observed that LPS up‐regulated FPR2 expression in trophoblast cells, which was consistent with the discovery of increased FPR2 expression in the placenta of CAM patients. The possible reason is that LPS combined with the trophoblast membrane receptor TLR4 initiates the inflammatory response signal pathway and at the same time up‐regulates the expression of FPR2 in trophoblast to promote its combination with endogenous anti‐inflammatory molecules balances, avoiding excessive inflammation.

Furthermore, we observed that LPS inhibits the activity of PPARγ and activate NF‐κB, which can be defused by RvD1. Meanwhile, the PPARγ blocker eliminated the effect of RvD1. It has been reported that ω‐unsaturated fatty acids, the natural ligand of PPARγ, which can inhibit the activation of NF‐κB induced by LPS in human kidney cells through PPARγ‐dependent signalling.^46^ In addition, the preventive application of RvD1 partially corrected the premature birth of LPS‐induced pregnant mice and improved the live birth rate. These results indicated that RvD1 may have an anti‐inflammatory effect through the PPARγ/NF‐κB pathway via combining to FPR2. However, Krishnamoorthy et al ^15^ transfected HEK‐293 cells with PPARγ and found that RvD1 did not activate PPARγ directly. We assume that RvD1 activates PPARγ through interaction with FPR2.

These results indicate that FPR2 plays an important protective role in inhibiting the development of inflammation in the early stages of infectious diseases in pregnancy. RvD1, via combining FPR2, inhibited excessive inflammatory response in trophoblast. Although our research suggests that the inhibitory effect of FPR2/RvD1 in the trophoblast inflammation model is achieved by activating PPARγ, as well as blocking the NF‐κB signalling pathway, the exact mechanism of the interaction between FPR2 and PPARγ requires further research and discussion. RvD1 and its precursor unsaturated fatty acids could be used as potential preventive and therapeutic medicines to improve the immunity of pregnant women and high‐risk pregnant women with bacterial infections.

## CONFLICT OF INTEREST

The authors confirm that there are no conflicts of interest.

## AUTHOR CONTRIBUTIONS

**Anna Li:** Project administration (lead). **Lin Zhang:** Data curation (lead). **Junxia Li:** Project administration (supporting). **Zhenya Fang:** Investigation (equal). **Shuxian Li:** Methodology (equal). **Yanjie Peng:** Data curation (supporting). **Meihua Zhang:** Conceptualization (equal). **Xietong Wang:** Conceptualization (equal).

## Supporting information

Fig S1

## Footnotes

1 Correction Statement: Correction added on 27 November 2020 after first online publication: The results section has been corrected in this version.

2 Correction Statement: Correction added on 27 November 2020 after first online publication: Figure 1 and its caption have been corrected in this version.
