# Supplementary figures and images for: Effect of RvD1/FPR2 on inflammatory response in chorioamnionitis
Source: J Cell Mol Med. 2020 Oct 6;24(22):13397–407. doi: 10.1111/jcmm.15963 (PMC7701521; doi:10.1111/jcmm.15963)

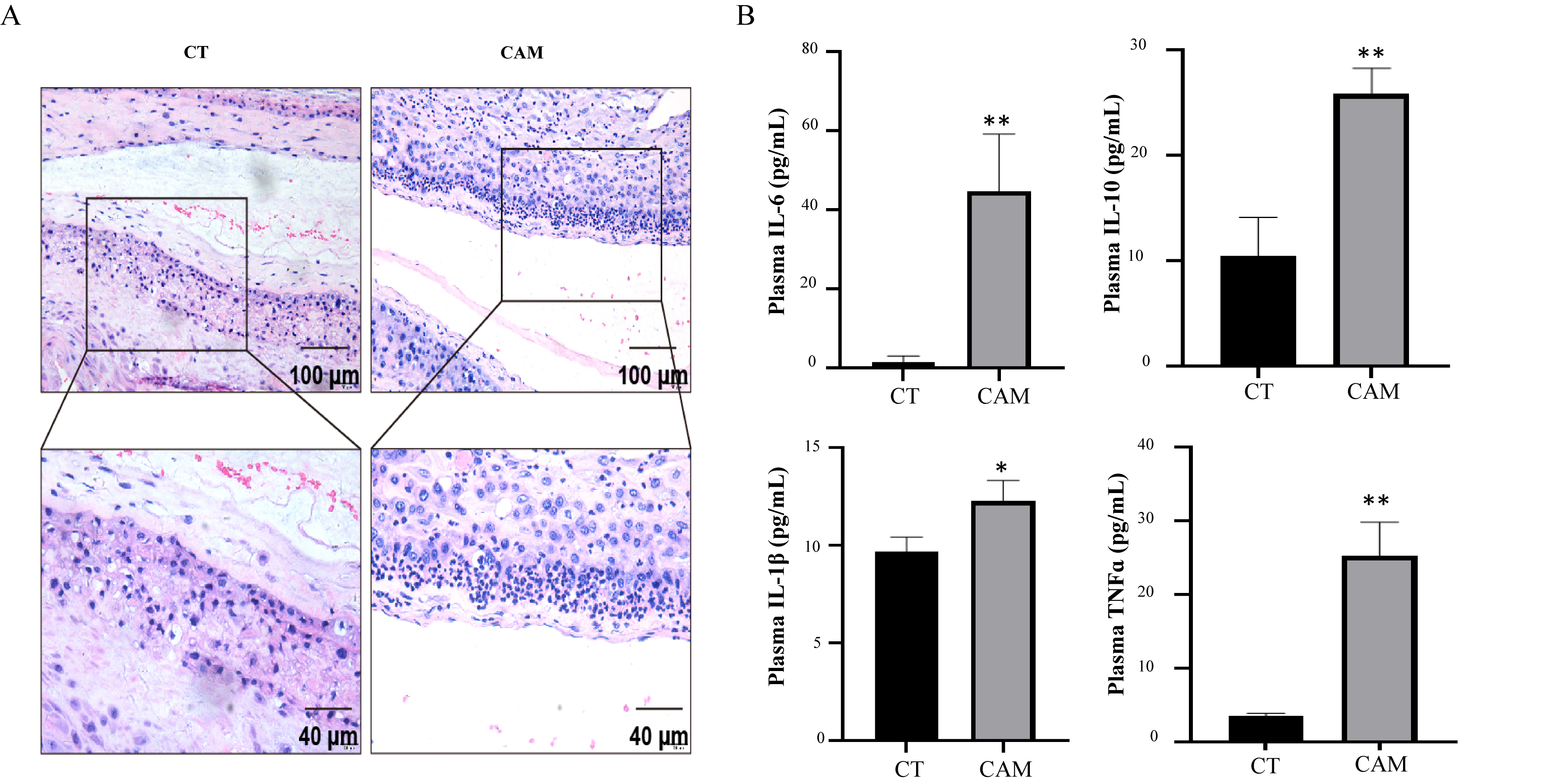

Supplement: Supplementary file 1 — Fig S1 [file JCMM-24-13397-s001.tif]
